# Salicylic Acid and Salicylate of Soda in the Treatment of Acute Rheumatism

**Published:** 1878-02

**Authors:** 


Salicylic Acid and Salicylate of Soda in the Treatment of Acute Rheumatism.—In the treatment of acute rheumatism with salicylic acid my experience extends over but one case, and in this the results were by no means satisfactory. Its failure in this instance may have been due, however, to the unusual malignancy (if I may apply this term) of the case. I will give a report of the case farther on. I was led to discontinue the use of salicylic acid and substitute for it the salicylate of soda in the treatment of this disease by having seen Prof. H. Kohler, of Halle, quoted as authority for the statement that "all the remote therapeutic effects of this remedy may be obtained by administering its salt—the salicylate of soda." The idea occurred to me that the salicylate would be even *better* than the acid, from the fact that in its use we would get the benefit not only of the salicylic acid, but also of whatever virtue there is in the alkaline plan of treatment. Sydenham, I believe it was, stated that the best treatment for inflammatory rheumatism was *six weeks*. Many high authorities since the days of Sydenham have doubted the usefulness of any medication for the *cure* of the disease, recommending only the use of opiates and liniments for the relief of sleeplessness and pain, and the use of placebos, such as peppermint-water (see Flint's Practice,) otherwise leaving the disease to the *vis medicatrix naturæ*.

The cases I have to report are as follows:

CASE I.—On the 10th of March, 1877, I was called to see A. C., a little girl some eight years of age. She had a high fever, severe headache, some nausea, and complained of pain in all of her limbs. Malarial fevers were prevailing in her neighborhood to some extent at that time; and although the family did not know of her having had a chill, I supposed the case to be one of remittent fever, and gave her a mercurial purgative and full dose of quinine. On the 11th, in the night, I was sent for to see her again. Found that the mercurial had acted well, and that the quinine had produced distinct tinnitus aurium; still her fever was higher, and she complained of great pain in elbows, wrists and knees. On making an examination I found that the joints were enlarged and quite hot. I saw at once that I had a case of acute articular rheumatism. I put her immediately on the salicylic acid treatment, giving her seven-and-a-half-grain doses every four hours. I also ordered for her a dose of the sulphate of morphine combined with nitrate of potash, every three or four hours until she was relieved from pain. In spite of this treatment the disease continued to increase in severity; so that by the 14th the pain in her joints, except when she was under the influence of the morphine, was intense, and she could not be moved without suffering the greatest agony. The dose of the acid was now increased to ten grains. No effect, however, was produced upon the disease, and the case continued with increasing severity until the 20th, when she succumbed.

In this case not only the joints were effected, but the *muscles* also became involved, as did the conjunctiva; so that during the last days of the patient's illness her abdomen was distended from swelling of the abdominal muscles and probably inflammation of the peritoneum; also her eyes inflamed and her face so greatly swollen that no one who knew her in health could have recognized in her distorted features those of the same person. Dr. S. C. Edmunds and Dr. R. T. Clark saw this case with me several times.

Case II.—W. B., a little girl seven years of age, was taken ill on September 5, 1877. I was called to see her on the 6th. She had considerable fever, furred tongue, bowels constipated, and pain in limbs, but no swelling in joints. Malarial history. Gave her five grains of calomel with four of Dover's powder, and prescribed for her twenty-four grains of sulphate of quinine in eight doses, one to be given every four hours. I saw her no more until the 10th, when I was called upon to visit her again. Learned that she was greatly benefited by the prescription I made at my first visit, but to-day I found her pulse to be eighty-eight to the minute, skin moist; her left elbow and wrist were considerably swollen, as well as hot and painful. I prescribed for her salicylate of soda in fifteen-grain doses, one to be given every four hours. She began to improve within six hours, and I had the satisfaction of learning two days afterward that her rheumatism had entirely disappeared. She had no return of the symptoms.

Case III.—November 20, 1877, visited C. C.; joints hot and swollen, pulse ninety-six to the minute, skin moist, bowels somewhat constipated. Gave a mercucial purgative and ordered a half ounce of salicylate of soda in eight powders, one to be given every three hours. On the 23d her husband informed me that she was entirely free from pain, and was able to sit up or walk about the room. On the evening of the 24th, however, the joints of her right arm and leg were again inflamed and quite painful. I had the above prescription refilled, and before she had finished taking it she was entirely free from pain, and the swelling was gone. The symptoms returned no more, and she soon regained her usual strength.

CASE IV.—On December 28th of last year, I was called to see Miss M. D. Was told that she had been confined to her bed for fifteen days, most of the time suffering acutely with inflammatory rheumatism. During this time she was under the treatment of Dr. D., an excellent physician of this town, and I presume of course had the benefit of the best treatment known for her disease before the introduction of salicylic acid and its salts. Dr. D.'s business engagements outside of the profession were such that he could not attend to the case; hence it fell under my care. The joints of all her limbs were red, hot, swollen and extremely painful, she stated. She also complained of great pain in the region of the heart. The joints of the left side were affected somewhat worse than those of the right, but they were all so much involved, that she could not be induced to move either hand or foot or change her position in bed. Profuse perspiration was a prominent symptom in the case. I made for her the following prescription :

℞.—Sodæ salicylatis, . . ℥ss.

Div. in chart, . . . . No. viij.

S.—Give a powder every four hours.

This prescription relieved her of the *pain* from which she was suffering almost as effectually as opium would have done; and at my next visit, twenty-four hours afterward, I found her very weak, of course, but entirely recovered from all symptoms of rheumatism, except slight swelling of the articulations. I continued the same prescription, except that the interval between the doses was lengthened to six hours, until the 30th, when, the swelling having disappeared and the natural suppleness of the joints having returned, I discontinued all treatment except tonics for the improvement of the appetite and strength. She felt no symptoms of her disease, and did well in every particular until the 6th of January, when inflammation again appeared in the left wrist and in the carpal and metacarpal joints of the left hand, and the pulse became accelerated to eighty-six beats to the minute. I had her resume the salicylate in doses of twenty-five grains every four hours for twenty-four hours, when the symptoms had again disappeared. She took twenty-five grains three times a day for some three days more, when she was able to resume her duties as matron of a female college. Since then she has been in the enjoyment of her usual health, and has felt no symptoms of rheumatism.

Case V.—On January 4th of this year was called to see L. H., a colored woman some twenty-five years of age. Found her suffering with all the symptoms of acute rheumatism, worst in the joints of her right arm, which it was impossible for her to move without suffering great pain. I prescribed for her the salicylate in doses of half a drachm every four hours. Two days later her fever had disappeared and the inflammation was gone from all the joints except the right wrist. I had this joint bathed twice a day with a stimulating liniment, and continued the salicylate in doses of twenty grains every six hours for some four days, by the end of which time she had entirely recovered.

Case VI.—On the 15th of January, this year, I was called to see Mrs. A. J., whom I found suffering severely with pain in the joints of all her limbs. She was an extremely corpulent woman, and I could not detect any swelling of the joints, though she thought they were somewhat enlarged; they were certainly very hot. I diagnosed acute rheumatism and prescribed the salicylate in doses of thirty grains every four hours. This case yielded more slowly to the salicylate than any other in which I have used it. The medicine nauseated her somewhat, and she could not be prevailed upon to take it with any regularity. When she was suffering severely, however, she would take a dose of the medicine, and it was followed by almost immediate relief of pain—as she said herself, "just as if she had taken a dose of morphine." She continued to take the medicine in this way until the 21st of the month, when, as she was no better except when under its influence, I

persuaded her to take a dose of the medicine regularly every six hours, in order to eradicate the disease entirely from her system. She took it for two days just as I directed, and at the end of that time she pronounced herself well, and she has continued so up to the present writing.

My success with salicylate of soda in acute rheumatism justifies me, I believe (in so far as I *can* be justified in forming conclusions from the small number of cases in which I have used it), in stating with reference to it what Dr. T. McLayan, in the London *Lancet*, and quoted in the *American Practitioner* of 1876, states of salicin, viz: . . "Given at the commencement of the attack, it seems to arrest the course of the malady as effectually as quinia cures an ague or ipecacuanha a dysentery;" and, I may add, it cures rheumatism much more effectually than ipecac. does dysentery in my practice; also, that "relief of pain is always one of the earliest effects produced." So much is this the case as applied to salicylic acid that in no instance where I have used this remedy have I had occasion to administer a single dose of opium or any other medicine of that class.

In the only case (Case IV) in which I began this remedy after the first few days of the attack, the patient had been ill fifteen days, and its results were as satisfactory as in any of the cases in which its use was begun "at the commencement of the attack."—*Louisville Medical News.*

